# Study of the glial cytoarchitecture of the developing olfactory bulb of a shark using immunochemical markers of radial glia

**DOI:** 10.1007/s00429-021-02448-9

**Published:** 2022-01-07

**Authors:** A. Docampo-Seara, E. Candal, M. A. Rodríguez

**Affiliations:** 1 Departamento de Bioloxía Funcional, Centro de Investigación en Bioloxía (CIBUS), Universidade de Santiago de Compostela, 15782 Santiago de Compostela, Spain; 2 UCL Institute of Ophthalmology, University College London, London, UK

**Keywords:** Development, Olfactory bulb, GFAP, GS, BLBP, Olfactory ensheathing cells, Sharks

## Abstract

During development of the olfactory bulb (OB), glial cells play key roles in axonal guiding/targeting, glomerular formation and synaptic plasticity. Studies in mammals have shown that radial glial cells and peripheral olfactory glia (olfactory ensheathing cells, OECs) are involved in the development of the OB. Most studies about the OB glia were carried out in mammals, but data are lacking in most non-mammalian vertebrates. In the present work, we studied the development of the OB glial system in the cartilaginous fish *Scyliorhinus canicula* (catshark) using antibodies against glial markers, such as glial fibrillary acidic protein (GFAP), brain lipid-binding protein (BLBP), and glutamine synthase (GS). These glial markers were expressed in cells with radial morphology lining the OB ventricle of embryos and this expression continues in ependymal cells (tanycytes) in early juveniles. Astrocyte-like cells were also observed in the granular layer and surrounding glomeruli. Numerous GS-positive cells were present in the primary olfactory pathway of embryos. In the developmental stages analysed, the olfactory nerve layer and the glomerular layer were the regions with higher GFAP, BLBP and GS immuno-reactivity. In addition, numerous BLBP-expressing cells (a marker of mammalian OECs) showing proliferative activity were present in the olfactory nerve layer. Our findings suggest that glial cells of peripheral and central origin coexist in the OB of catshark embryos and early juveniles. These results open the path for future studies about the differential roles of glial cells in the catshark OB during embryonic development and in adulthood.

## Introduction

Within the central nervous system, the study of the olfactory system has become a focus of attention because it presents sustained neurogenesis and continuous axonal outgrowth from the olfactory epithelium, ensuing plasticity in the olfactory bulb (OB) (Su and He 2010; Roet and Verhaagen 2014; Lim and Alvarez-Buylla 2016; Calvo-Ochoa et al. 2021).The olfactory bulb (OB) is a primary sensory centre located in the telencephalon, which receives input about odours detected by olfactory receptor neurons (ORNs) located in the olfactory epithelium in the nasal cavity. ORN axons project to the OB, where they terminate in huge globular synaptic structures referred to as glomeruli; these glomeruli are functional units where odour inputs are first processed within the central nervous system (Mori et al. 1999).

In mammals, glial cells are involved in diverse processes (axonal outgrowth/targeting, formation/stabilization of olfactory glomeruli) during OB development (Doucette 1990; Valverde et al. 1992; Gonzalez et al. 1993; Gonzalez and Silver 1994; Bailey et al. 1999; Puche and Shipley 2001; Au et al. 2002; Tolbert et al. 2004; Doengi et al. 2009; Roux et al. 2011; Su et al. 2013; Amaya et al. 2015a, b; Terni et al. 2017). Approaching the study of the development of the OB glial system in mammals and other non-mammalian animal models has proven challenging because of the difficulty in defining the different types of glial cells, since many of these types share morphology, location and/or molecular markers. In the developing forebrain of mammals, cells expressing glial markers are first observed at the onset of neurogenesis, when neuro-epithelial cells divide asymmetrically and give rise to neurons and to apical radial glial cells (RGCs). Apical RGCs are progenitor cells with apico-basal polarity and cell bodies located in the ventricular zone. They express glial markers and present extended radial processes that contact with the apical (ventricular) and basal (pial) surfaces of the walls of the developing brain, and therefore play an important role as a scaffold for neuronal migration. In mammals, apical RGCs are transient; these cells disappear in postnatal stages by symmetric self-consuming divisions that produce either immature neurons or glial cells with which they share many glial markers, such as astrocytes and oligodendrocytes (Paridaen and Huttner 2014), except for a few brain areas, where apical RGCs give rise to basal progenitors with cell bodies located out of the ventricular zone, which are maintained as adult neural stem cells throughout life (Götz 2013). In mammals, ependymal cells, though expressing glial markers, does not have radial morphology. In contrast, in anamniotes (fishes and amphibians), the radial ependymoglia represent the predominant glial cell type in the brain during embryogenesis and in adulthood and they express glial markers similar to that found in apical RGCs (Kálmán and Gould 2001; Cuoghi and Mola 2009; Allen and Lyons 2018). The molecular and morphological similarities between ependymoglia and RGCs in anamniotes make it difficult to discriminate between these cell types in late development (see Docampo-Seara et al. 2018).

In the developing OB of rats, RGCs appear at embryonic day 16–17 and its organization pattern and morphology change as development progresses. These cells express glial markers similar to those expressed by cortical RGCs as the intermediate filament glial fibrillary acid protein (GFAP) (Bailey et al. 1999; Puche and Shipley 2001) and brain lipid-binding protein (BLBP) (Barraud et al. 2018). In the OB of mammals, the number of RGCs decreases postnatally, while the number of astrocytes increases progressively. Several morphological subtypes of astrocytes have been reported in the OB of rodents using antibodies against GFAP (Bailey and Shipley 1993; Chiu and Greer 1996; Bailey et al. 1999; Olude et al. 2014, 2015; Klein et al. 2020). In addition, other RGCs marker such as the enzyme glutamine synthetase (GS; involved in the conversion of glutamate to glutamine) has been also reported to be located in astrocytes in the OB of mouse (Okere and Kaba 2000). To our knowledge, similar studies about the OB glial system of non-mammalian vertebrates during embryogenesis are lacking, although there are some reports in later stages in reptiles and amphibians, i.e., ependymoglial cells express GFAP in the OB of juvenile reptiles (Lazzari and Franceschini 2006) and in the frog olfactory system from tadpoles to adults (Huang et al. 2005). Although the expression pattern of GFAP has been studied in the brain of adult cartilaginous fishes (Wasowicz et al. 1999; Kálmán and Gould 2001; Ari and Kálmán 2008a, b), descriptions about the GFAP expression pattern in the OB are not included in these studies. Moreover, as far as we are aware, data about the OB glial system in embryos and early postnatal life are lacking in cartilaginous fishes.

A class of peripheral glial cells termed olfactory ensheathing cells (OECs) is also required for the formation of olfactory glomeruli. These cells are exclusive of the peripheral and central olfactory system and represent a morphologically and molecularly heterogeneous population (Su and He 2010). OECs migrate toward the OB along the axon bundles of the olfactory nerve and express neurotrophic factors and cell adhesion molecules implicated in ORN axonal outgrowth (Su and He 2010; Roet and Verhaagen 2014). In addition, OECs are related with the continued regeneration of ORNs throughout life. Based on their capability to sustain regeneration, OECs transplantation therapies have been used for repairing neural injury in the central and peripheral nervous system (for review see: Reshamwala et al. 2019; Su and He 2010). In mammals, OECs share many molecular markers with RGCs, astrocytes and Schwann cells, including BLBP, GFAP and S100β protein (Valverde et al. 1992; Astic et al. 1998; Miller et al. 2010; Huilgol and Tole 2016). However, information about the OECs in non-mammalian vertebrates is limited (adult and metamorphic amphibians: Huang et al. 2005; Lazzari et al. 2016; adult teleosts: Lazzari et al. 2013, 2014). Previous studies of our group during development of the catshark have reported the presence of GFAP-immunoreactive putative OECs in the peripheral olfactory system (Quintana-Urzainqui et al. 2014a), but the expression of other glial markers at late embryonic stages or in juveniles has not been examined so far.

Most of the studies about the OB glia are focused on mammals and little is known about its development and role in fishes. The gnathostome crown group of vertebrates is subdivided in osteichthyans (bony fishes plus tetrapods) and chondricthyans (cartilaginous fishes), which diverged from a common ancestor about 525 million years ago (Blair and Hedges 2005). Embryological studies and whole-genome analysis in elasmobranchs indicate that this group of fishes is key for understanding the early evolution of jawed vertebrates (Gillis and Shubin 2009; Venkatesh et al. 2014; Rodríguez-Moldes et al. 2017; Hara et al. 2018).

The olfactory system of cartilaginous fishes is essential for survival; sharks have a very developed sense of smell and their OBs are considerably large with respect to the size of the brain (Yopak et al. 2015). Although the OBs of sharks have a laminar structure, they do not present the six layers described in mammals. Three main layers can be observed in the catshark (from outside to inside): the olfactory nerve layer, the glomerular layer and the granular layer (Smeets et al. 1983). Mitral cells, the main OB projection cells in catshark (Yáñez et al. 2011), are diffusely distributed between the glomerular and granular layer, unlike in mammals.

With the purpose of shedding light on the development of glial cells of the OB in an evo-devo context, we studied the expression of three radial glia markers (GFAP, BLBP and GS) in catshark embryos and early juveniles using immunohistochemical techniques. We also evaluated the proliferative capacity of glial cells by combining immunohistochemistry against the proliferating cell nuclear antigen (PCNA) with these glial markers. Additionally, we used double immunofluorescence techniques with antibodies against GS and a marker of primary olfactory fibres (trimeric G protein Gα0 subunit) to study the relationship between the central and peripheral glia and olfactory glomeruli.

## Materials and methods

### Experimental animals

In the present study, we analysed 15 embryos of *S. canicula* at stages 31 (S31) to 34 (S34) and 5 juvenile animals. Embryos were staged by their external features according to Ballard et al. (1993). Embryos were provided by the Marine Biological Model Supply Service of the CNRS UPMC Roscoff Biological Station (France) and the Oceanographic Observatory of Banyuls sur Mer (France). Juveniles were kindly provided by the aquarium of O Grove (Galicia, Spain). Catsharks were raised in seawater tanks under standard conditions of temperature (15–16 °C), pH (7.5–8.5) and salinity (35 g/L) and suitable measures were taken to minimize animal pain and discomfort. All procedures were made in accordance to the guidelines established by the European Communities Council Directive of 22 September 2010 (2010/63/UE) and by Spanish Royal Decree 1386/2018 for animal experimentation and were approved by the Ethics Committee of the University of Santiago de Compostela.

### Tissue processing

Embryos were deeply anesthetized with 0.5% tricaine methane sulfonate (MS-222; Sigma, St. Louis, MO) in seawater and separated from the egg yolk before fixation in cold 4% paraformaldehyde (PFA) in elasmobranch phosphate buffer [EPB: 0.1 M phosphate buffer (PB) containing 1.75% urea, pH 7.4] for 48–72 h. Juveniles were deeply anesthetized with MS-222 and then perfused intra-cardially with elasmobranch Ringer´s solution (see Ferreiro-Galve et al. 2012) followed by 4% PFA in EPB. Brains of perfused juveniles were removed and post-fixed in the same fixative for 24–48 h at 4 °C. Subsequently, they were rinsed in PB saline (PBS), cryo-protected with 30% sucrose in PB, embedded in OCT compound (Tissue Tek, Torrance, CA), and frozen with liquid nitrogen-cooled isopentane. Parallel series of transverse sectios (16–18 μm thick) were cut on a cryostat and mounted onto Superfrost Plus slides (Menzel-Glasser, Madison, WI, USA).

### Immunohistochemistry

For immunohistochemistry, sections were pre-treated with 0.01 M citrate buffer pH 6.0 for 30 min at 90 °C for heat-induced epitope retrieval and allowed to cool for 20 min at room temperature (RT). Sections were rinsed in 0.05 M Tris-buffered saline pH 7.4 (TBS) for 5 min, treated with 10% H_2_O_2_ in TBS for 30 min at RT to block endogenous peroxidase activity, rinsed again in TBS for 5 min and incubated with the primary antibodies (see Table 1) for 15 h at RT. Sections were rinsed three times in TBS for 10 min each, and incubated in the appropriate HRP-coupled secondary antibody (see Table 1) for 1 h at RT. All dilutions were made with TBS containing 15% normal goat serum (Millipore, Billerica, MA), 0.2% Triton X-100 (Sigma) and 2% bovine serum albumin (BSA, Sigma) and incubations were carried out in a humid chamber. Then, sections were rinsed three times in TBS for 10 min each and the immune complex was developed with 0.25 mg/ml diaminobenzidine tetrahydrochloride (DAB, Sigma) and 0.00075% H_2_O_2_ in TBS pH 7.4, or with SIGMAFAST™ 3.3-DAB tablets as indicated by the manufacturers. Finally, the sections were dehydrated and cover-slipped.

**Table 1:** Primary and secondary antibodies used

Primary antiboDY	Source	Working dilution	Secondary antibody	Source	Working dilution
PCNA	Monoclonal mouse anti-PCNA Sigma Cat. Nº. P8825	1:500	488-conjugated donkey anti-mouse	Alexa Fluor Molecular Probes, Eugene, OR	1:200
GFAP	Polyclonal rabbit anti-GFAP Dako Cat. Nº. Z033429	1:500	Goat anti-rabbit HRP-coupled	Dako, Glostrup, Denmark	1:200
GS	Monoclonal mouse anti-GS Millipore Cat. Nº. MAB302	1:500	Goat anti-mouse HRP coupled	Dako, Glostrup, Denmark	1:200
BLBP	Polyclonal rabbit anti-BLBP Millipore Cat. Nº. ABN14	1:300	546-conjugated donkey anti-rabbit	Alexa Fluor Molecular Probes, Eugene, OR	1:200
Gα0	Polyclonal rabbit anti- Gα0 Santa Cruz Biotech Cat. Nº. sc-387	1:400			

### Double immunofluorescence

After heat-induced epitope retrieval, sections were rinsed in TBS for 5 min, incubated with primary antibodies (see Table 1) for 15 h at RT and rinsed three times in TBS (see above). Then, sections were incubated in the appropriate combination of fluorescent dye-labelled secondary antibodies (see Table 1) for 1 h at RT. All dilutions were made with TBS containing 15% normal donkey serum, 0.2% Triton X-100 and 2% BSA. All incubations were carried out in a humid chamber. Sections were rinsed three times in TBS (10 min each), rinsed in distilled water (30 min), dried for 30 min at 37 °C and mounted with MOWIOL 4–88 (Calbiochem/Merk KGaA, Darmstadt, Germany) or with Vectashield mounting medium for fluorescence with DAPI (Vector, Burlingame, California). Information about the primary and secondary antibodies is included in Table 1.

### Control and specificity of antibodies

The PCNA monoclonal antibody has been previously used to label proliferating cells in a number of vertebrate species, including the brain and olfactory system of *S. canicula* (i.e. Ferreiro-Galve et al. 2010; Quintana-Urzainqui et al. 2014a; Quintana-Urzainqui et al. 2015). The specificity of the antibodies against glial markers (GFAP, GS and BLBP) has also been tested by western blot in catshark brain extracts (Docampo-Seara et al. 2018), showing they stain a protein band of the appropriate MW. Omission of these primary antibodies from the immunostaining procedures led to unstained sections. The Gα0 antibody was used here as an anatomical marker of the primary olfactory system, as previously used in this catshark (Ferrando et al. 2009).

### Imaging

Fluorescent sections were photographed with a Leica TCS-SP2 spectral confocal scanning microscope with an appropriate combination for blue and green excitation lines. Confocal images were acquired separately for each laser channel with steps of 2 μm along the z-axis, and collapsed images were obtained with the LITE software (Leica). Some sections were photographed with an Olympus DP70 colour digital camera fitted on an Olympus AX70 fluorescence microscope. Light field images were obtained with an Olympus DP71 colour digital camera fitted on an Olympus BX51 microscope. Images were adjusted for contrast, brightness and intensity, and plates were prepared using Corel Draw X7.

## Results

**Fig. 1:**
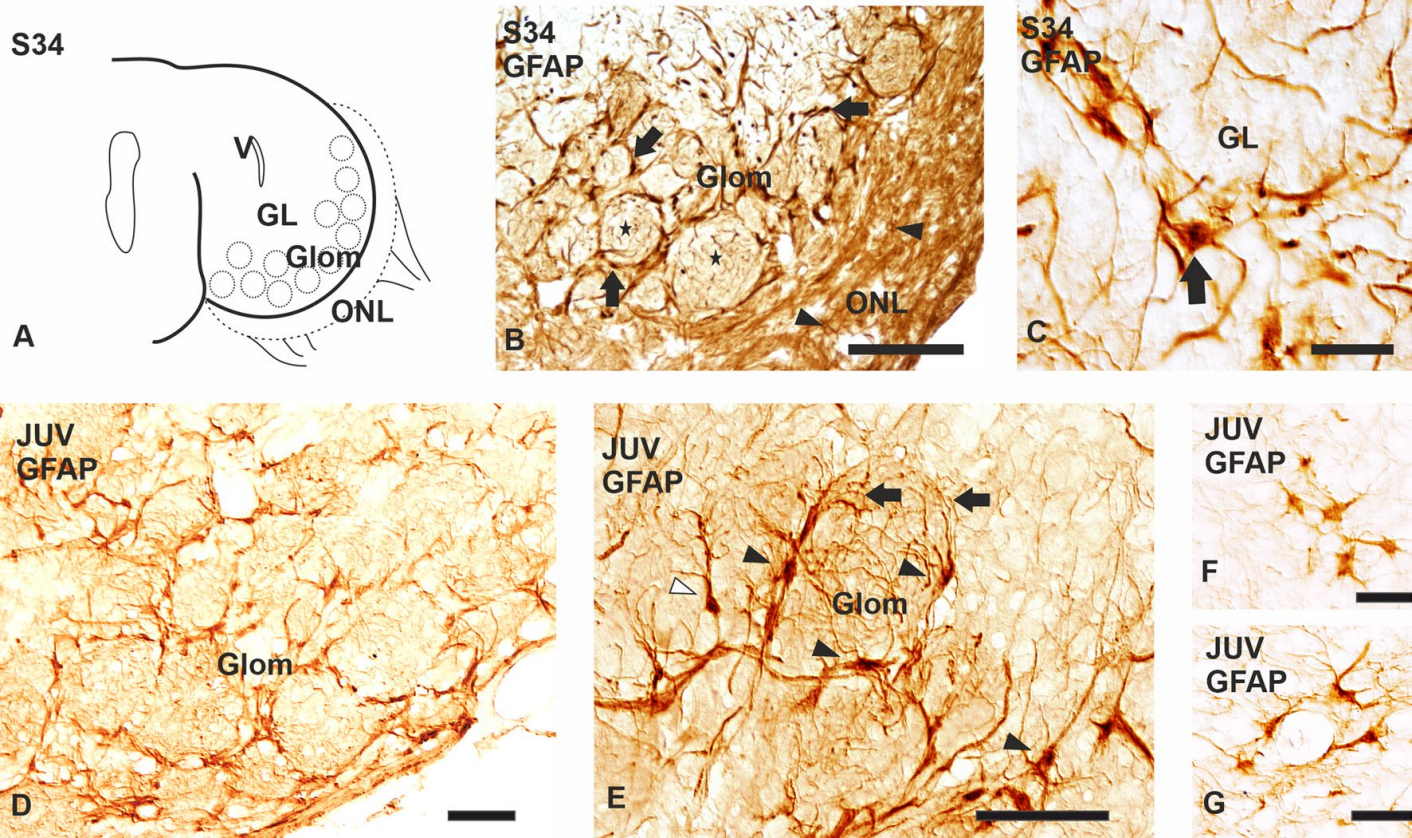
Schema (**A**) and photomicrographs of transverse sections of the OB of *Scyliorhinus canicula* showing the expression of GFAP in embryos (**B**, **C**) and juveniles (**D**–**G**). **B**, **C** Photomicrographs of a stage 34 (S34). **B** Low magnification photomicrograph showing GFAP-positive cells surrounding glomeruli (arrows), as well as thin positive processes inside the glomerular neuropil (stars). Note also extensive GFAP positivity in the olfactory nerve layer, and a few GFAP-positive cells (arrowheads). **C** High magnification photomicrograph showing GFAP immunoreactivity in astrocyte-like cells in the granular layer (arrow in **C**) and also perivascular immunoreactivity. **D**–**G** Transverse sections of the OB of juveniles. **D**, **E** Photomicrographs showing numerous GFAP-positive glial cells surrounding glomeruli forming shell-like structures. Positive glial cells in the glomerular layer show highly branched processes that invade the neuropil of glomeruli (arrows in **E**). Note that these cells exhibit bipolar morphology (black arrowheads) with tufted processes (white arrowhead). **F**–**G** Photomicrographs showing GFAP-positive astrocyte-like cells in the granular layer (**F**); these cells are usually seen surrounding blood vessels (**G**). *GL* granular layer, *Glom* glomerular layer, *ONL* olfactory nerve layer, *V* ventricular zone. Scale bars: 500 µm (**D**), 100 µm (**B**), 50 µm (**B**, **E**), 25 µm (**C**), 10 µm (**F**, **G**)

In catshark embryos, the olfactory bulb primordium (OP) protrudes in lateral portions of the telencephalic hemispheres at the transition from stages S30 to S31 and, at S31, the OP becomes a well-defined lateral protrusion. While no clear cell layering is appreciable at this stage, incipient glomeruli or protoglomeruli begin to appear in the distal portion of the OP. At S32, protoglomeruli are more evident and the basic layering of the olfactory bulb (OB) becomes appreciable from outside to inside: the olfactory nerve layer, the glomerular layer (protoglomeruli) and the granular layer (Fig. 1A). Mitral cells, which in the OB of mammals and in other vertebrates form a distinct cell layer, are rather diffusely distributed between the glomerular and granular layers in the catshark OB. From S32 onwards, the OB grows considerably, and the olfactory nerve layer, glomerular layer and granular layer become easily recognizable. For further information about the OB development in the catshark, see Quintana-Urzainqui et al. (2014a, 2015).

### Expression of radial glia markers (GFAP, BLBP and GS) in the OP of intermediate embryos (S31), and OB of late embryos (S32-S34) and juveniles

The expression of radial glia markers (GFAP, BLBP and GS) was detected in the OB of *Scyliorhinus canicula* from S31 embryos onwards. Numerous GFAP-, BLBP-, and GS-immunoreactive cells lining the OB ventricle with the typical morphology of RGCs with stained radial processes (ependymal cells or tanycytes: Horstmann 1954) were present in embryos and posthatching juveniles. This pattern was similar to that previously reported in the lateral telencephalic ventricles (Docampo-Seara et al. 2018). In all developmental stages analysed, immunoreactive glial cells were observed in all layers of the OB.

At S31, GFAP immunoreactivity was observed in glial processes accompanying the bundles of primary olfactory axons, which was identified as OECs in a previous study in the catshark (Quintana-Urzainqui et al. 2014a), and this persists in the olfactory nerve of late embryos and juveniles (data not shown). At the same developmental stage, GFAP-immunoreactive glial processes were detected in the OP. The amount of immunoreactive glial processes increases in S32 and S33 embryos, but GFAP-positive glial cell bodies were not appreciable in the OB until S34 (Fig. 1A–C). A few GFAP-immunorrective glial cells were observed in the olfactory nerve layer, where numerous immunoreactive processes were also present (Fig. 1B). The glomerular layer showed the highest levels of GFAP immunolabelling, whereas glial cells with a bipolar morphology were observed surrounding olfactory glomeruli, forming a glomerular shell-like structure; some of the positive glial cells showed either thin or thick branched processes. In addition, numerous GFAP-immunoreactive glial processes were visualized within the glomerular neuropil (Fig. 1B). In the granular layer, numerous GFAP-immunoreactive glial cells with a stellate morphology and short radially oriented processes were observed. Many of them showed a perivascular location and remind us astrocyte-like cells (Fig. 1C). The GFAP expression pattern observed in S34 embryos is similar to that observed in juveniles (Fig. 1D–G).

**Fig. 2:**
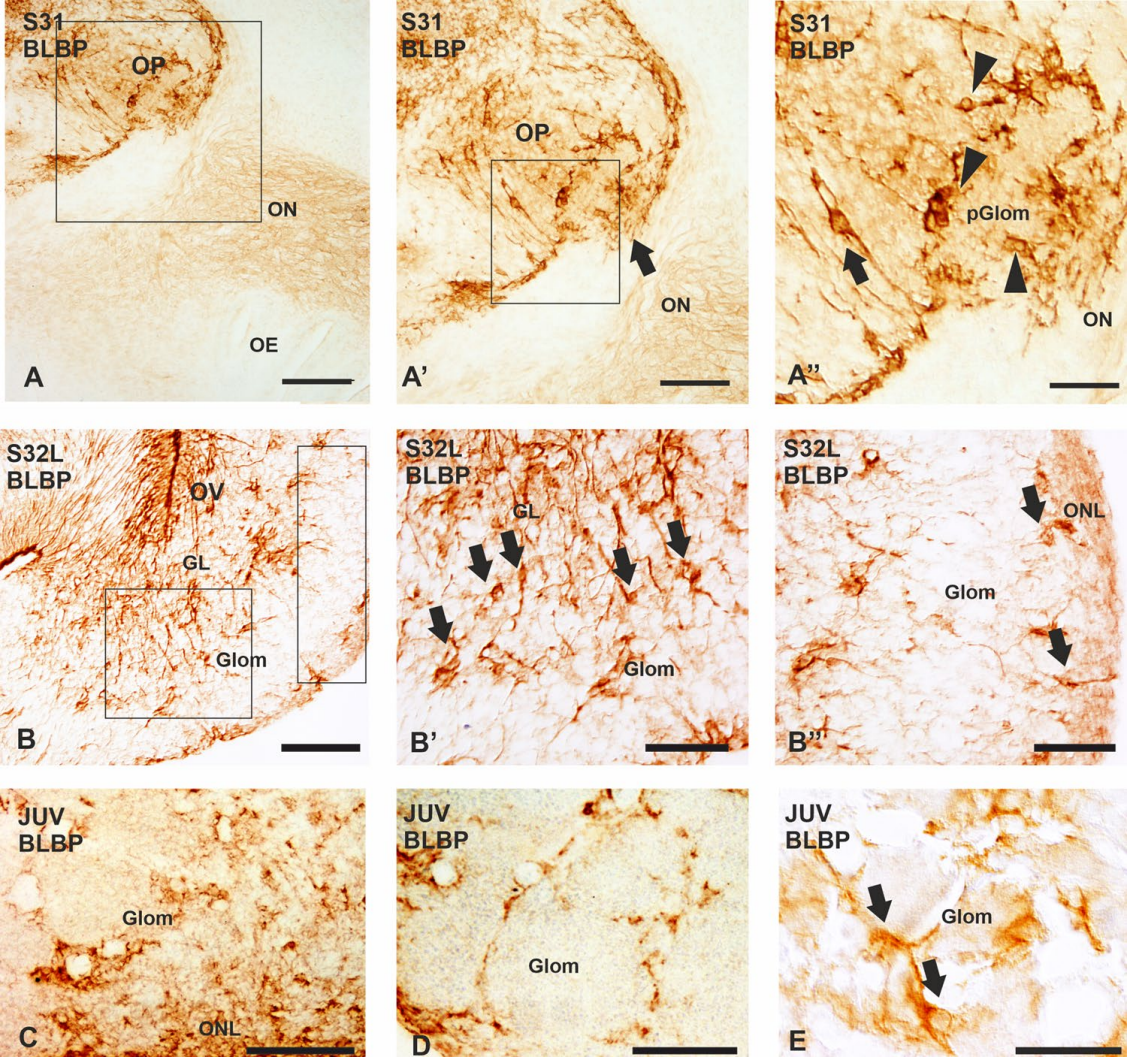
Photomicrographs of transverse sections of the OB of *Scyliorhinus canicula* showing the expression pattern of BLBP in embryos (**A**–**B”**) and juveniles (**C**–**E**). **A**–**A**’’ Photomicrograph and details of the olfactory bulb primordium of a stage 31 (S31). Note faint BLBP immunoreactivity in the ON and intense BLBP-positive cells with different morphologies in the region where the ON meets the OP (arrow in A’), as well as in adjacent regions (arrow and arrowheads in A”). **B**–**B**’’ Photomicrograph and details of the OB showing the expression of BLBP at late stage 32 (S32L). Scattered BLBP-positive cells are located in the outer and inner regions of the glomerular layer, in contrast with the high amount of BLBP-positive cells observed in the granular layer (arrows in **B**’). Note the presence of BLBP-positive cells (arrows in **B**´´) and processes in the ONL. **C**–**D** Transverse sections of the OB of juveniles. BLBP is abundantly expressed in the glomerular layer and the ONL (**C**). In the glomerular layer, BLBP-positive cells surround glomeruli (**D**). **E** Photomicrograph showing some BLBP-positive cells in the glomerular layer (arrows). *GL* granular layer, *pGlom* protoglomeruli, *Glom* glomerular layer, *OE* olfactory epithelium, *ON* olfactory nerve, *ONL* olfactory nerve layer, *OP* olfactory primordium, *OV* olfactory ventricle. Scale bars: 100 µm (**A**, **B**, **C**) 50 µm (**A**’, **B**’, **B**’’, **D**), 25 µm (**A**’’, **E**)

The expression of BLBP and GS was first detected in the OP in S31 embryos. At this developmental stage, the olfactory nerve showed a faint BLBP expression (Fig. 2A–A’). Numerous BLBP-positive glial cells were appreciable from the entrance of the olfactory nerve, where the bundles of primary olfactory axons end in compact protoglomerular fields (arrowheads in Fig. 2A’’). Scattered BLBP-positive bipolar glial cells with radial processes close to the meningeal surface were seen in the OP (arrow in Fig. 2A’’). At S32, BLBP immunoreactivity increases; the highest staining intensity was observed in glial processes of the granular layer, in contrast with the low BLBP immunoreactivity observed in glial processes of the glomerular layer (Fig. 2B). Numerous BLBP-positive glial cell bodies with a bipolar morphology were observed throughout the granular layer, as well as in regions close to the glomeruli and olfactory nerve layer (arrows in Fig. 2B–B´´). The BLBP expression pattern observed in S34 embryos is similar to that observed in S32 embryos. However, in juveniles, immunoreactive structures were not found in the granular layer, although BLBP-immunoreactive glial cell bodies and processes were seen surrounding the glomeruli. The glomerular neuropil was devoid of BLBP immunoreactivity (Fig. 2C-E). Numerous BLBP-immunoreactive glial cells were visualized in the olfactory nerve layer, which seem to be more numerous in its outer part (Fig. 2C).

**Fig. 3:**
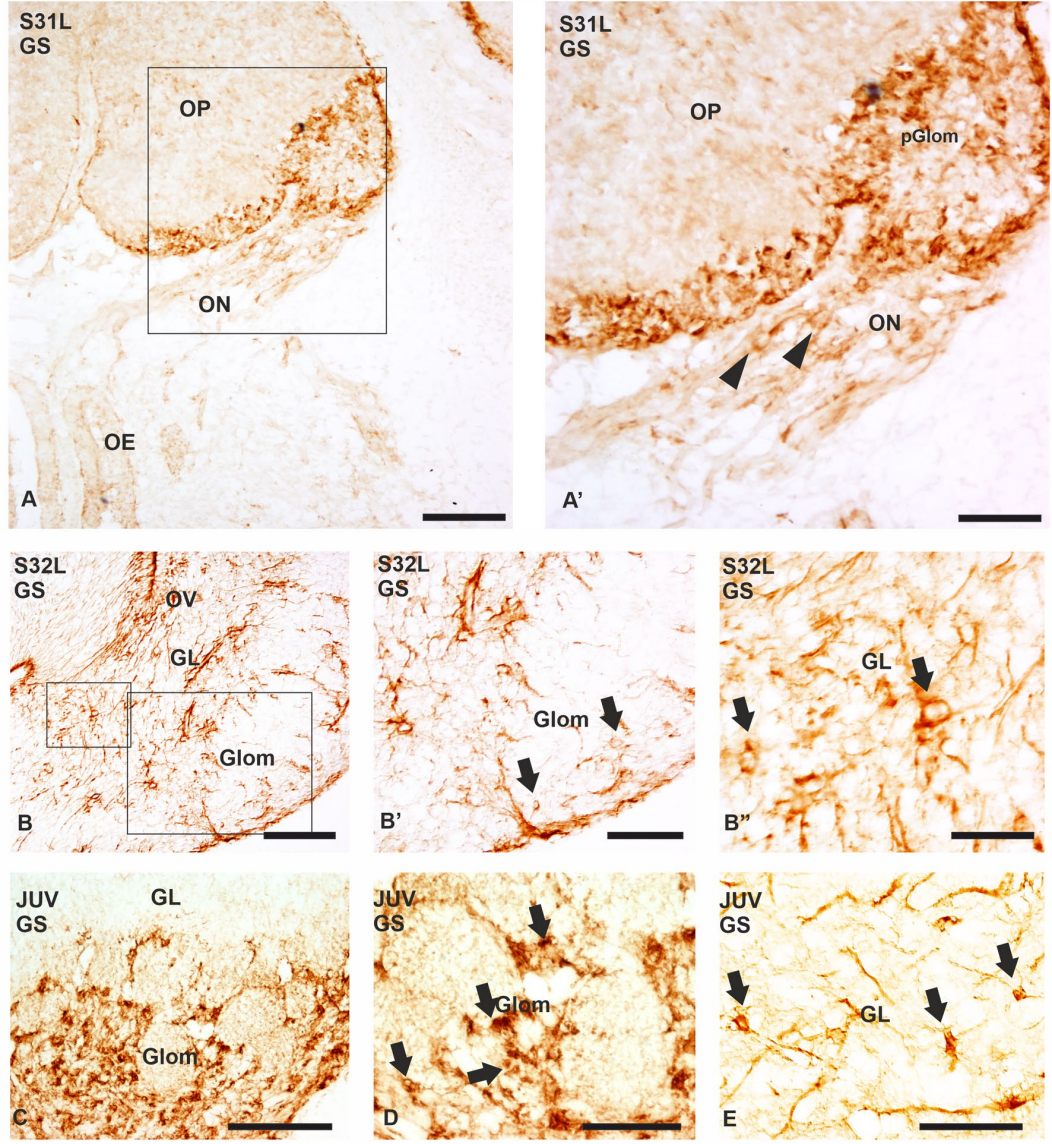
Photomicrographs of transverse sections of the OB of *Scyliorhinus canicula* showing the expression of GS in embryos (**A**–**B**’’) (**B**) and juveniles (**C**–**E**). **A**–**A**’ Photomicraph (**A**) and detail (**A**’) of the OP in a late stage 31 (S31L) showing GS-immunoreactive cells in the protoglomeruli. Note some positive cells in the ON (arrows). **B**–**B**’’) Photomicrograph and details showing the expression pattern of GS at late stage 32 (S32L). The glomerular layer shows scattered GS-positive cells (arrows in **B**’), in contrast to the granular layer (arrows in **B**’’). **C**–**D** Transverse sections of the OB of juveniles. At these developmental stages dense aggregates of GS-positive cells surround the glomeruli (**C**, and arrows in **D**) and small highly branched cells are widely distributed in the granular layer (arrows in **E**). *GL* granular layer, *pGlom* protoglomeruli, *Glom* glomerular layer, *OE* olfactory epithelium, *ON* olfactory nerve, *ONL* olfactory nerve layer, *OP* olfactory primordium, *OV* olfactory ventricle. Scale bars: 100 µm (**A**, **B**, **C**) 50 µm (**A**’, **B**’, **D**, **E**), 25 µm (**B**’’)

GS immunoreactivity was first detected in S31 embryos. Faintly immunolabelled glial cells were present in the olfactory nerve and dense GS-immunoreactive aggregates (glial cell bodies and processes) were present in the region where primary olfactory axons terminate forming protoglomeruli (Fig. 3A–A’). The GS expression pattern in S32 and S34 embryos is similar. At S32, numerous GS-immunoreactive glial cell bodies and processes were located in the granular layer, but immunoreactive glial cells were scarcer in the glomerular layer and olfactory nerve layer (Fig. 3B–B’’). In juveniles GS-positive glial cells and processes were scarce in the granular layer, but were numerous surrounding the glomeruli, (Fig. 3C and arrows in D). In contrast to the large glomerular glial cells, GS-immunoreactive glial cells observed in the granular layer are small and exhibit a bipolar morphology; in addition, GS containing cells with a stellate morphology were also present in the granular layer (arrows in Fig. 3E).

### Double immunofluorescence for GS and GFAP or BLBP

At S32, numerous GS-positive glial cell bodies and a large amount of GFAP-immunoreactive processes were observed. Numerous glial cells expressed BLBP, and its expression pattern was similar to that observed with GS immunohistochemistry at the same stage. In juveniles, the periglomerular glial cells positive for these glial markers were abundant and apparently showed morphological differences with regard to their branching, soma or even their disposition surrounding glomeruli. Double immunofluorescence against GS/GFAP and GS/BLBP was performed in S32 embryos and in juveniles.

**Fig. 4:**
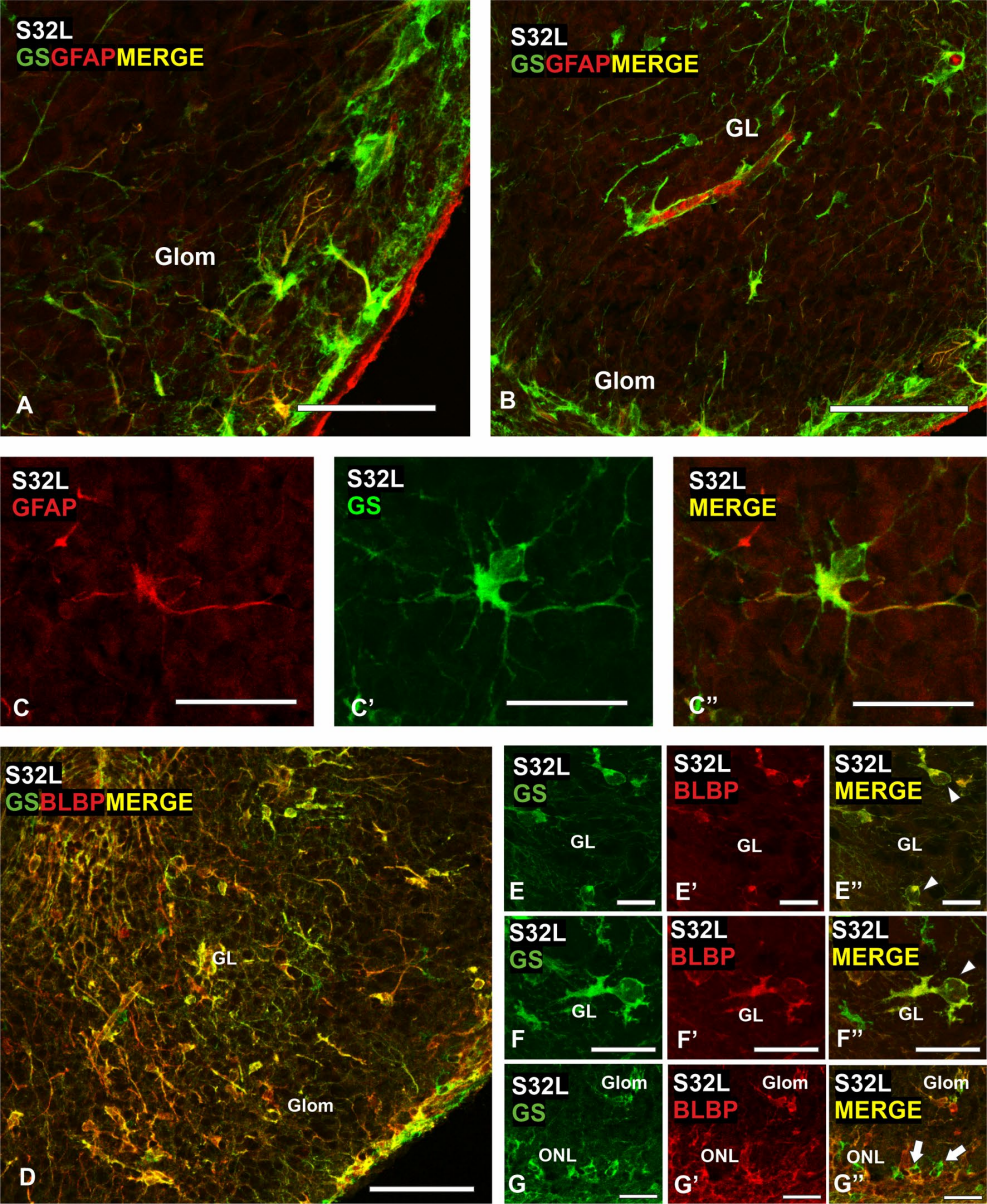
Photomicrographs of the OB showing double immunolabeling for GS and GFAP (**A**–**C**’’), and GS and BLBP (**D**–**G**’’) in a late stage 32 (S32L). **A**, **B** Photomicrogaphs of a S32 embryo, showing immunolabeled processes in the glomerular layer (**A**) and in the granular layer (**B**). Note colocalization of both glial markers in some processes. **C**–**C**’’ Detail of a GFAP/GS double-labelled cell showing a particular morphology. **D**–**G**’’ Photomicrographs of the OB and details showing double immunofluorescence for GS and BLBP. Note that most of glial cells in the granular layer are double-labelled (arrowheads in **E**’’ and **F**’’), in contrast with the ONL where glial markers do not colocalize (arrows in **G**’’). GL, granular layer; Glom, glomerular layer. Scale bars: 200 µm (**D**), 100 µm (**B**), 50 µm (**A**), 10 µm (**C**–**C**’’, **E**–**G**’’)

Although at S32 glial cell bodies were GFAP-negative, there were numerous GFAP-positive (GFAP +) processes that were also GS + (Fig. 4A–B). Strikingly, the combination of both markers allows us to detect some isolated cells with a particular morphology (Fig. 4C–C’). These rare cells possess GS + /GFAP negative (−) cell bodies with a large protrusion giving rise to several GS + /GFAP + processes. On the other hand, GS/BLBP double immunofluorescence showed that both glial markers are co-expressed by glial cells in the glomerular and granular layers (Fig. 4D–F’’). However, at the level of olfactory nerve layer, GS + /BLBP − and BLBP + /GS − cells were appreciable (Fig. 4G–G’’’).

**Fig. 5:**
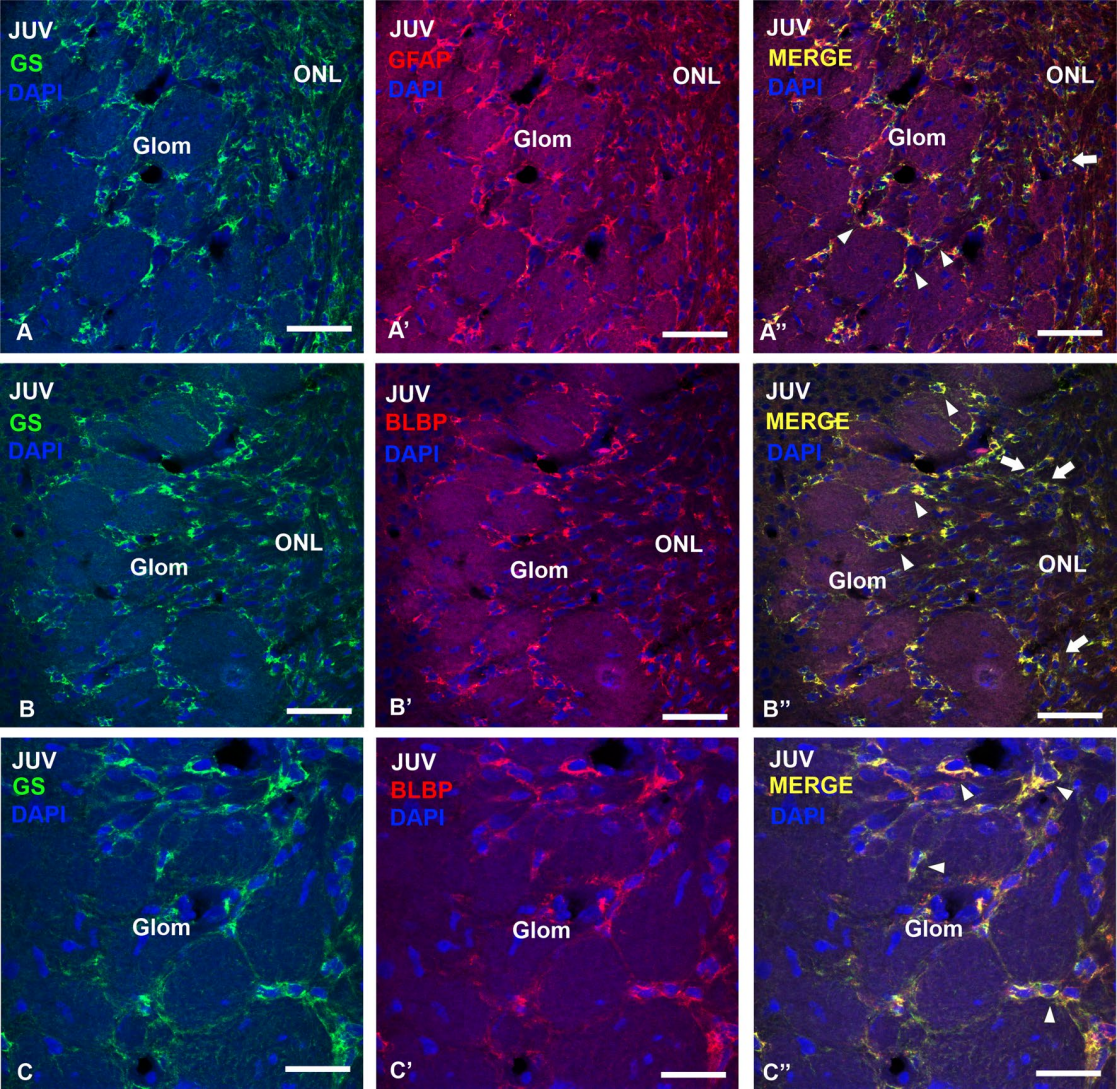
Photomicrographs of the OB of *Scyliorhinus canicula* juveniles showing double immunolabeling for GS and GFAP (**A**–**A**’’), GS and BLBP (**B**–**C**’’) counterstained with DAPI. **A**–**A**’’) Photomicrographs of the OB showing double-labelled GS/GFAP cells in the Glom (arrowheads) and ONL (arrow). Note that glial markers are mostly co-expressed. (**B**–**C**’’) Photomicrograph and details of the OB showing that GS-positive cells in the Glom (arrowheads in **B**’’ and **C**’’) and ONL (arrows in **B**’’) are also BLBP-positive. *GL* granular layer, *Glom* glomerular layer, *ONL* olfactory nerve layer. Scale bars: 50 µm (**A**, **A**’, **A**’’, **B**, **B**’, **B**’’), 25 µm (**C**, **C**’, **C**’’)

Double immunofluorescence against GFAP/GS and BLBP/GS combined with DAPI staining was also performed in juveniles. Interestingly, both GFAP/GS (Fig. 5A–A’’) and BLBP/GS (Fig. 5B–C’’) double immunofluorescence showed that periglomerular glial cells co-expressed all these glial markers. In addition, co-expression of GFAP and GS was observed in cell bodies and/or processes in the olfactory nerve layer (Fig. 5A–A’’), which was also seen in the granular layer. On the other hand, BLBP and GS were co-expressed in the same cells in the olfactory nerve layer (Fig. 5B–B’’).

### Double immunofluorescence for BLBP/PCNA and GS/Gα0

**Fig. 6:**
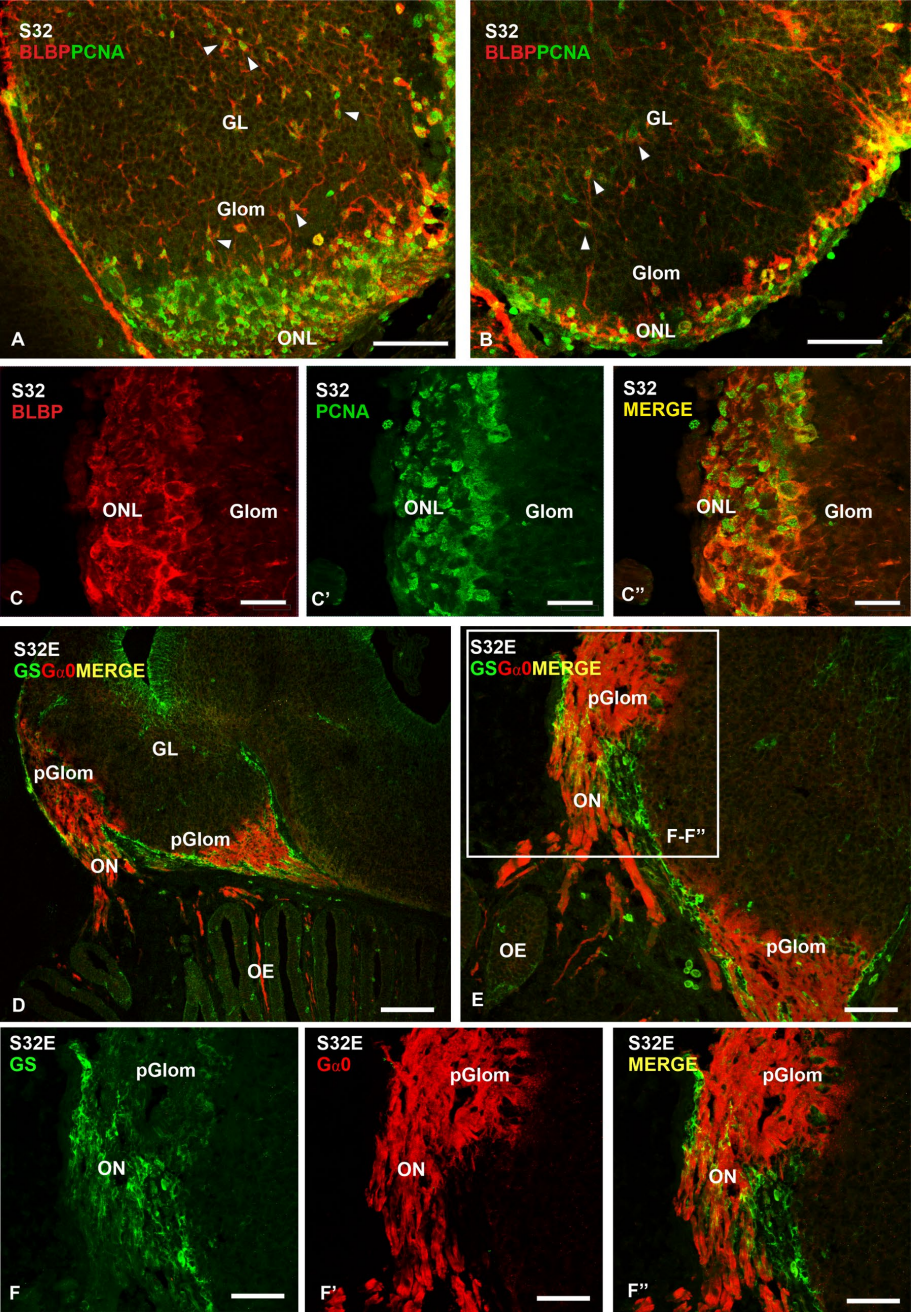
Photomicrographs of different sections of the OB of *Scyliorhinus canicula*  embryos showing double immunofluorescence for PCNA and BLBP at stage 32 (S32) (**A**–**C**’’), and GS and Gα0 at early stage 32 (S32E) (**D**–**F**’’). **A**, **B** Panoramic views at different levels of the OB showing that BLBP and PCNA co-localize in cells of all layers (arrowheads). Note the high amount of proliferating BLBP-positive cells in the ONL (details at higher magnification, **C**–**C**’’). (**D**–**E**) Photomicrographs (**D**–**E**) and details (**F**–**F**’’) showing double immunofluorescence for GS and Gα0. Note numerous GS-positive cells in the region where the primary olfactory fibres meet in protoglomerular fields (**D** and **E**), and also extending along the ON (**F**–**F**’’). *GL* granular layer, *pGlom* protoglomeruli, *Glom* glomerular layer, *OE* olfactory epithelium, *ON* olfactory nerve, *ONL* olfactory nerve layer. Scale bars: 500 µm (**D**), 200 µm (**A**, **B**, **E**), 50 µm (**F**, **F**’, **F**’’) 25 µm (**C**, **C**’, **C**’’)

The presence of numerous BLBP + and GS + cells in the olfactory nerve layer and olfactory nerve during development raises the possibility that they are OECs. We performed double immunofluorescence for BLBP and the proliferation marker PCNA, in order to determine whether these cells show proliferative activity; and double immunofluorescence for GS and Gα0 (a marker of the primary olfactory fibres) in order to determine if these cells envelop the olfactory nerve in S32. We observed that BLBP and PCNA were coexpressed in the same cells (Fig. 6A, B). Positive cells for both markers were observed in the glomerular and granular layers. However, the highest amount of PCNA-positive glial cells was observed in the olfactory nerve layer, and apparently all of them seem to be double-labelled (Fig. 6C–C’’). On the other hand, double immunofluorescence for GS and Gα0 showed GS-positive glial cells extending along the olfactory nerve (primary olfactory fibres are positive for Gα0), as well as in the region where the primary olfactory axons end in compact protoglomerular fields, which also showed Gα0 immunoreactivity; expression pattern of Gα0 observed in the present work was previously described by Quintana-Urzainqui et al. (2014a) in catshark embryos (Fig. 6D–F’’).

## Discussion

The present work represents the first developmental study of the glial system focused on the OB of a cartilaginous fish using immunohistochemical markers of radial glia and cell proliferation. We have analysed the expression of GFAP, BLBP, GS and PCNA in the developing OB of *Scyliorhinus canicula* from its onset at intermediate embryonic stages (S31) until late developmental stages (from S32 to hatching) (developmental stages according to Ballard et al. 1993), as well as in post-hatching (early) juveniles. The early development of the olfactory epithelium and nerve, together with the terminal nerve, has been previously described (Quintana-Urzainqui et al. 2014a, b). By comparison with the development of neuronal components of these nerves, the onset of glial markers in the OB is delayed.

**Fig. 7:**
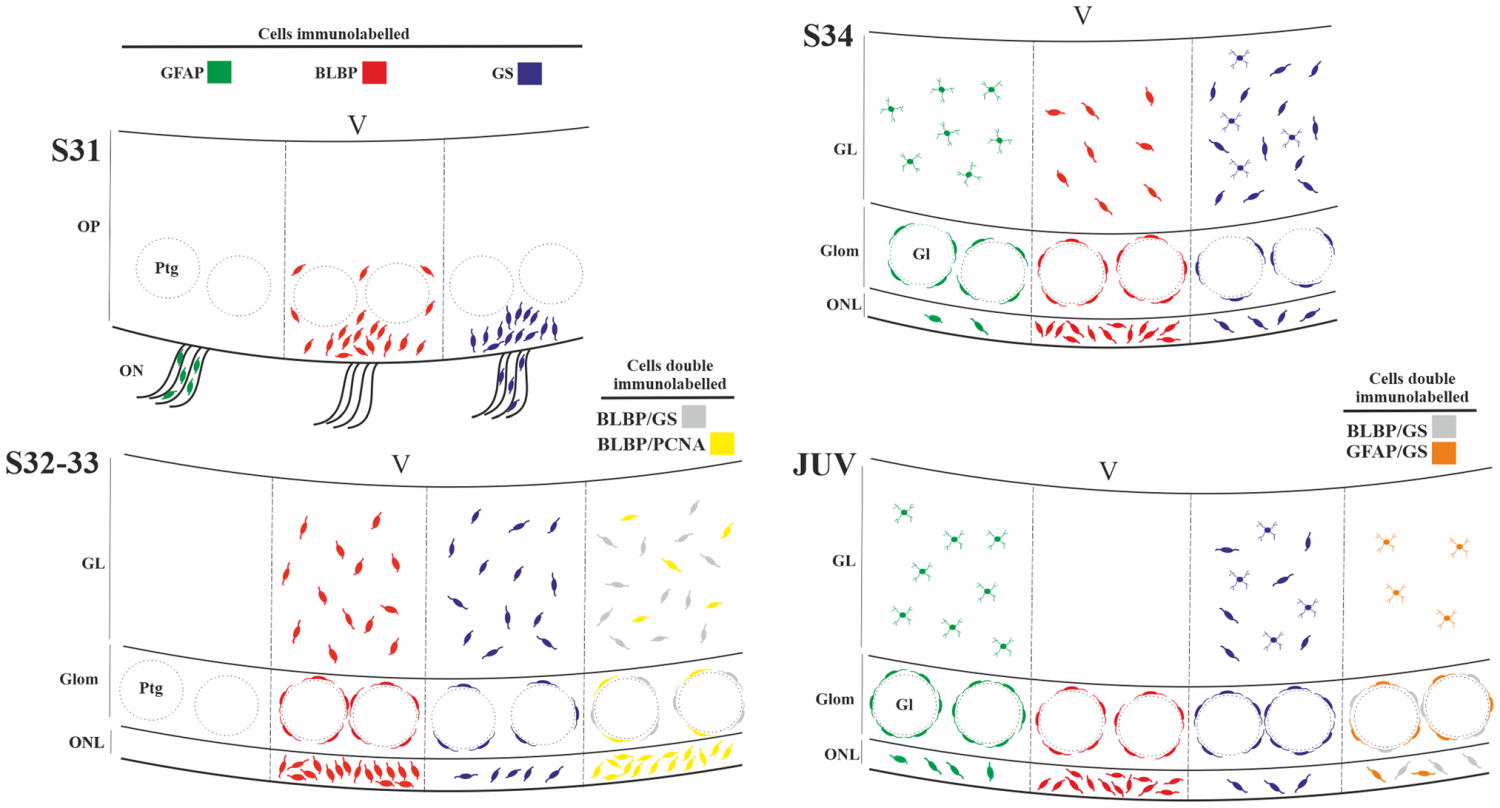
Schema summarizing the distribution pattern of GFAP, BLBP and GS containing cells in the OP and ON of embryos (S31), OB of embryos (S32, S33, S34) and juveniles of *Scyliorhinus canicula*. Double immunolabelled cells for BLBP/GS and BLBP/PCNA in a S32, and double-labelled cells for BLBP/GS and GFAP/GS in juveniles are also included in the schema. Immunoreactive fibres and ventricular glia are not represented in the schema. *Gl* glomeruli, *GL* granular layer, *Glom* glomerular layer, *OP* olfactory primordium, *ON* olfactory nerve, *ONL* olfactory nerve layer, *pGlom* protoglomeruli, *V* ventricle

We have showed that GFAP, BLBP and GS expression in nuclei and/or cell processes appear at the same developmental stage, when the OP becomes recognizable in the catshark. All markers used label glial cells with the typical morphology of RGCs and astrocyte-like cells in late embryos; this morphological diversity was also observed in all layers of the OB of juveniles, in which GFAP, BLBP and GS were mainly expressed in the glomerular and olfactory nerve layers where the three markers were expressed by the same glial cells. In late embryos, numerous BLBP-expressing cells exhibited proliferative capacity (PCNA immunoreactivity) in all layers of the OB, although the highest amount of PCNA-positive cells was observed in the olfactory nerve layer. In addition, numerous GS-immunoreactive glial cells were present in the protoglomeruli and the olfactory nerve. The chronological development of GFAP, BLBP and GS containing cells observed in the OP and OB of the catshark is summarized in Fig. 7. These results and their possible functional implications are discussed below in a comparative evolutionary developmental context.

### GFAP, BLBP and GS expression reveals glial cells with different morphology in the developing OB of *Scyliorhinus canicula*

In the developing OB of catshark, GFAP immunoreactivity was detected from mid-developmental stages (S31) and its expression persists in the late embryonic period (S32-34), as well as in post-hatching juveniles. Numerous GFAP-immunoreactive cells (present results) with the morphology of RGC lined the OB ventricle of embryos and post-haching juveniles (ependymal cells or tanycytes: Horstmann 1954), and their apical processes are organized as previously reported in the telencephalic hemispheres (Docampo-Seara et al. 2018), but no changes in organization were observed during development. Similarities and differences can be noted between the glia of catshark and mammals. In rodents, GFAP is also expressed in cells of the OB in the late embryonic period (Astic et al. 1998; Bailey et al. 1999; Puche and Shipley 2001), but the morphology of RGCs changes during development. At E14, RGCs show similar morphology to cortical RGCs and its processes appear arranged parallel to each other (Rakic 1972), whereas at E16 processes of RGCs branch and form distinct types of glial plexuses throughout the OB.

In the catshark, GFAP immunoreactivity was first observed in S31 embryos along the olfactory nerve, as well as in the region where the olfactory nerve establish contact with the OP. Numerous immunoreactive processes were found in the olfactory nerve layer from S32 onwards, which is in agreement with previous findings in catshark (Quintana-Urzainqui et al. 2014a) and is similar to that reported in rats, where intense GFAP staining is noted along the fascicles of olfactory nerve fibres at E16 (Astic et al. 1998). Numerous GFAP-expressing cells with different morphologies were first observed in the glomerular and granular layer of the catshark OB from S34 onwards. In the developing OB of mammals, first GFAP-expressing cells are seen at E19 in rat (Bailey et al. 1999) and at E17.5 in mouse (Puche and Shipley 2001). Our results in late embryos/juveniles of catsharks have showed that the glomerular layer shows the highest levels of GFAP immunoreactivity, which is in agreement with results in rodents (Bailey et al. 1999; Puche and Shipley 2001) and reptiles (juveniles; Lazzari and Franceschini 2006). In the catshark, GFAP-expressing cells with bipolar morphology and highly branched processes form shell-like structures around glomeruli (present results). Cells with a similar morphology called “radial astrocytes” surround the glomeruli in the embryonic OB of rat (Bailey et al. 1999). In the catshark OB, scattered GFAP-positive astrocyte-like cells were found in the granular layer from S34 onwards (present results). Although OB astrocytes expressing GFAP are scarce in rodents just after birth, their number increases postnatally as far as that of RGCs decreases (Bailey et al. 1999; Puche and Shipley 2001); In the mature OB of rodents, several morphological categories of astrocytes have been reported according to their degree of branching and disposal of processes, and astrocytes also undergo morphological changes with age (Klein et al. 2020). The glomerular layer presents diverse morphological subtypes of astrocytes, some of them exhibiting layer specificity, and each astrocyte associates with a single glomerulus (Bailey and Shipley 1993; Bailey et al. 1999; Chiu and Greer 1996). Numerous GFAP-positive cells and processes have been also described in all layers of the OB in the African giant rat, also showing regional and molecular heterogeneity (Olude et al. 2014, 2015). Clonal analysis of astrocytes after electroporation in embryonic mice has revealed both layer specificity and glial heterogeneity inside each OB layer (García-Marqués and López-Mascaraque 2017).

Although astrocyte-like cells are present in the catshark OB (present results), the morphological diversity seems to be much lower than in mammals. Interestingly, glial cells with astrocyte morphology are not present in the catshark telencephalic hemispheres during development or in juveniles (Docampo-Seara et al. 2018). On the other hand, studies in the adult brain of other cartilaginous fish and teleosts have evidenced important differences in distribution of astrocyte-like cells. Species of cartilaginous fishes, such as the ray *Torpedo *sp. and the skate *Raya *sp., show a large number of astrocytes in the adult forebrain and midbrain (i.e. Kálmán and Gould 2001; Ari and Kálmán 2008a, b), whereas in teleost fishes, astrocyte-like cells are mainly found in the optic nerve, rhombencephalon and spinal cord (reviewed by Cuoghi and Mola 2009). Together, these findings indicate that the distribution of astrocytes is not homogeneous in the brain within the same species, and that there are important interspecies differences in fishes.

BLBP is a marker of RGCs during cortical development in mammals (Götz 2013), but data about BLBP expression in the developing and mature OB are very scant throughout vertebrates. In S31 catshark embryos, BLBP-expressing cells were mainly observed at the olfactory nerve entrance in the OB, whereas in later embryos, both BLBP-positive ependymal cells and cells in the granular layer with morphology of RGCs were also present. After hatching, the expression of BLBP became restricted to the OB ependyma, glomerular layer and olfactory nerve layer. The expression pattern of BLBP is highly similar to that of GS in the OB of embryonic and juvenile catshark. In mice, BLBP is also expressed in the olfactory nerve layer on E16.5 (Barraud et al. 2018) and, in adults, GS-positive cells are found in the plexiform, mitral, and granule cell layers (Okere and Kaba 2000). However, unlike in the catshark, the glomerular layer of the mouse shows low amount of GS. BLBP-immunoreactive processes were also reported in the mature OB of a ray-finned fish, *Austrolebias charrua* (Rosillo et al. 2016).

#### BLBP and olfactory ensheathing cells (OECs) in the developing ON/OB of catshark

A notable result in the OB of catshark S32 embryos was that most of the BLBP-positive cells showed proliferative activity (i.e., they were PCNA-ir). It is striking the high amount of these proliferating cells found in the olfactory nerve layer and also associated to the olfactory nerve entrance (present results). In mammals, ORN axons in the olfactory nerve directed toward the glomerular layer are accompanied by a population of migratory cells collectively named migratory mass, which consists of several types of cell populations including neurons and OECs. OECs are a class of peripheral glia restricted to the olfactory system that is thought to be derived from either the olfactory placode (Miller et al. 2010) or the neural crest (Barraud et al. 2010). These OECs surround ORN axon fascicles in the olfactory nerve and olfactory nerve layer, and their number increase by mitotic divisions (Valverde et al. 1992; Au et al. 2002; Miller et al. 2010; Blanchart et al. 2011; Geller et al. 2017). OECs guide newly generated primary olfactory axons growing towards appropriate glomerular targets lifetime (Su and He 2010). OECs of the olfactory nerve layer show some cellular and molecular differences with the OECs derived from the lamina propia (Roet and Verhaagen 2014). Recent studies in rodents have shown that BLBP is a marker of OECs progenitors (Murdoch and Roskams 2007; Blanchart et al. 2011; Barraud et al. 2018), and this is in agreement with our findings in the catshark. Previous studies have also shown that OECs express GFAP in catshark (Quintana-Urzainqui et al. 2014a), mammals (Astic et al. 1998), amphibians (Huang et al. 2005; Lazzari et al. 2016) and teleosts (Lazzari et al. 2013, 2014). All these findings support the hypothesis of Quintana-Urzainqui et al. (2014a) that OECs were already present in the common ancestor of jawed vertebrates. The nature of the olfactory nerve glia in lampreys remains to be determined.

As far as we are aware, GS was not previously identified as a marker of OECs in mammals or other vertebrates, unlike that observed here in the catshark, despite they share expression of other molecular markers of astrocytes and Schwann cell. In rodents, GS was related with Schwann cell differentiation (Saitoh and Araki 2010; Su and He 2010). Our results in the catshark suggest that peripheral and central glial cells coexist in the OB of embryos and juveniles. Further studies are needed to elucidate the molecular identity of the OECs in the catshark, and to determine the progeny of the proliferative BLBP-positive cells detected in the OB.

### Putative implications of glia in the OB development in the catshark

ORNs are continuously replaced throughout life in rodents. Thus, the formation and maintenance of the olfactory system involve the correct interaction of the growing axons of ORNs with their appropriate targets in the OB lifetime; however, it is known that in the embryonic and postnatal life, numerous ORN axons misroute to inappropriate targets (Amaya et al. 2015a). In transgenic mouse embryos, RGCs positive for BLBP remove axonal debris from overextending axons (Amaya et al. 2015a, b), and other studies have shown that OECs phagocytise apoptotic olfactory nerve debris (Su et al. 2013). Our results have showed numerous BLBP-positive cells in the embryonic and postnatal OB of catshark, which similarly might also act as phagocytes. Further investigations are necessary to elucidate the actual roles of these cells in the OB.

Numerous investigations in mammals indicate that glia-neuron interactions play an important role in the organization of olfactory glomeruli, where olfactory odour information is primarily processed (Mori et al. 1999). Glomerular astrocytes detect neuronal activity (Roux et al. 2011) and express extracellular matrix molecules which provide boundaries for growing of primary olfactory axons (Doucette 1990; Gonzalez et al. 1993; Gonzalez and Silver 1994). Similarly, in the antennal (olfactory) lobe of insects, glial cells establish boundaries that define protoglomeruli, and the penetration of peripheral glial cells into the glomerular region is key in the glomerular formation (Tolbert et al. 2004). Besides, in mammals, during the glomerular formation in late embryonic stages, mitral cells change in shape and size and their dendrites undergo morphological modifications (Valverde et al. 1992; Bailey et al. 1999; Blanchart et al. 2006). Glomerular formation is initiated when olfactory axons contact dendrites in the OB. Then, cells from the migratory mass contact with the prospective glomerular layer surrounding protoglomeruli and, postnatally, glomeruli, letting these cells to transform in periglomerular astrocytes (Valverde et al. 1992). Our results have showed that glomeruli in the catshark are also composed of a glial shell-like structure from which numerous thin glial processes invade the neuropil glomerular, resembling the glial shell described in mammals. These similarities between mammals and the catshark led us to suggest that periglomerular glial cells in the OB of catshark are derived from putative OECs of the migratory mass and may play similar roles to those of mammals.

With regard the numerous GS-positive cells and processes observed in the granular and glomerular layers of embryonic and juvenile catsharks (present results), they are probably involved in the regulation of glutamate transmission at these places. In glutamatergic synapses, most of the glutamate released to the synaptic cleft is taken up by astrocytes, where is transformed to glutamine by the action of the enzyme GS (Battú et al. 2005). In the mammalian OB, GABA uptake from the synaptic cleft by astrocytes is also involved in neuron-glia signalling (Doengi et al. 2009). Previous studies in catshark embryos reveal that migratory GABAergic cells originated from the ventral telencephalon invade the OB to form granular cells (Carrera 2008; Quintana-Urzainqui et al. 2015). TH immunoreactive cells appear in the granular layer of the OB in S32 embryos and later in periglomerular regions (Carrera et al. 2012). The distribution of GS-positive processes in the catshark OB is related to sites where synaptic transmission between olfactory axons (glutamatergic), mitral cells (glutamatergic) and periglomerular and granule neurons (GABAergic) is expected to occur (Sueiro 2003; Ferrando et al. 2012), which also suggests that these OB glial cells are involved in synaptic plasticity.

## Conclusion

The present work represents the first developmental study in a cartilaginous fish focused on the glial cells of the OB, using different glial markers. The radial glia markers GFAP, BLBP and GS were first expressed in S31 embryos, and its expression persist in the postnatal OB. Cells with morphologies of RGCs (ependymal cells or tanycytes) and astrocyte-like cells were present in the OB, which reveals a higher glial cell diversity than in other catshark brain areas (Docampo-Seara et al. 2018). In late embryos and juveniles, numerous glial cells were observed surrounding the glomeruli. Numerous proliferating glial cells positive for BLBP (a marker of mammalian OECs), have been detected in the olfactory nerve layer in late embryos. In addition, GS-positive cells were present in the olfactory nerve in catshark embryos, and this enzyme colocalized with BLBP in the olfactory nerve layer of juveniles. These findings suggest the presence of both peripheral (OECs-derived) and central glial cells in the developing OB of *Scyliorhinus canicula*. The present work establishes a basis for future studies on the functional roles of glial cells in the developing olfactory system of sharks.

## Data Availability

The present manuscript titled “Study of the glial cytoarchitecture of the developing olfactory bulb of a shark using immunochemical markers of radial glia”, include images of optical and confocal microscopy from sections of the olfactory bulb of *Scyliorhinus canicula*. The material in the manuscript is not under consideration for publication elsewhere, has not been previously published and will not be submitted elsewhere for publication. All authors are sure that all data and materials included in the manuscript claims and comply with field standards, and raw imaging and microscopy data generated in this study can be requested from the authors upon reasonable request.
